# Effects of free range-of-motion upper limb exercise based on mirror therapy on shoulder function in patients after breast cancer surgery: study protocol for a randomized controlled trial

**DOI:** 10.1186/s13063-021-05789-2

**Published:** 2021-11-17

**Authors:** Ru-Zhen Yuan, Kun-Peng Li, Xiao-Lin Wei, Wei Zheng, Yi Ye, Ming-Yue Wang, Jie-Ting Jiang, Cai-Qin Wu

**Affiliations:** 1 School of Nursing, Shanghai University of Traditional Chinese Medicine, 1200 Cailun Road, Pudong New District, Shanghai, China; 2 Shanghai East Hospital Tongji University, Shanghai, China; 3 School of Rehabilitation, Shanghai University of Traditional Chinese Medicine, 1200 Cailun Road, Pudong New District, Shanghai, China; 4 Department of Galactophore, Longhua Hospital Shanghai University of Traditional Chinese Medicine, Shanghai, China; 5 Department of General Surgery, Fudan University Zhongshan Hospital, Shanghai, China

**Keywords:** Breast cancer survivors, Free range-of-motion upper limb exercise, Mirror therapy, Shoulder function

## Abstract

**Background:**

Shoulder function complications are common after treatment for breast cancer. Quite a few survivors still report a limited shoulder range of motion, even though the free range-of-motion upper limb exercise is helpful to restore shoulder function. Mirror therapy (MT) is a classical and effective rehabilitation technique to recover motor and sensory function for the limbs; in addition, studies have reported that MT has an influence on patients with shoulder functional dysfunction including increasing shoulder range of motion, improving shoulder function scores, and decreasing pain scores. Here, we describe a protocol of a randomized controlled trial to explore if free range-of-motion upper limb exercise based on MT has efficacy on shoulder function in survivors after surgery of breast cancer.

**Methods/design:**

This is a prospective, single-blind, two-arm randomized controlled trial. An estimated 70 participants will be randomly allocated to (1) the MT group or (2) the control group. The participants in the control group receive free range-of-motion upper limb exercise, and participants in the MT group will engage in free range-of-motion upper limb exercise based on MT. The intervention will start on the first day after surgery and be completed at 8 weeks after surgery. The primary outcome in this protocol is shoulder range of motion (ROM), while the Constant-Murley Score (CMS); Disability of the Arm, Shoulder, and Hand Questionnaire (DASH); Tampa Scale of Kinesiophobia (13-item TSK); visual analog scale (VAS); grip strength; arm circumference; and lymphedema are the secondary outcomes. Assessment will be conducted before allocation (baseline) and at 2 weeks, 4 weeks, and 8 weeks after surgery.

**Discussion:**

Based on the results that MT has an influence on shoulder function immediately after intervention in patients without nerve injury, this randomized controlled trial is to observe the efficacy of MT on shoulder function after a long-term intervention in breast cancer survivors. We look forward to the innovation of this study for both breast cancer rehabilitation and MT.

**Trial registration:**

Chinese Clinical Trial Registry (ChiCTR) ChiCTR2000033080. Registered on 19 May 2020

**Supplementary Information:**

The online version contains supplementary material available at 10.1186/s13063-021-05789-2.

## Background

Breast cancer is the most frequently diagnosed cancer among women throughout the world [1]; however, the 5-year survival for breast cancer is the highest among women’s cancer, and it is going up gradually [2]. Since both prevalence and survival rates continue to rise, breast cancer tends to become chronic. Similar to patients with chronic diseases, breast cancer survivors (BCS) have to fight the treatment-related complications that affect their health status and quality of life. Shoulder function complications are one of the most common sequelae following treatment for breast cancer, which can manifest soon after surgery or would appear many years later. Typical symptoms include a restricted shoulder range of motion (ROM), stiffness and weakness of the shoulder, and pain [3]. It is reported that there is a prevalence rate of 32.9% of a restricted shoulder ROM in patients at 1 year after axillary lymph node dissection and a prevalence rate of 23.2% in patients at 1 year after sentinel lymph node biopsy [4]. Pain and numbness are found to be 51.1% in BCS at 2 years after breast cancer surgery [5]. Shoulder function complications will adversely affect BCS in daily activity, medical burden, social life, and quality of life. It is limited to follow-up time; shoulder function complications persist much longer than it is reported in many studies. Hauerslev et al. reported that 25.7% and 30% BCS who are evaluated 11.7 years after surgery have decreased shoulder mobility and shoulder pain, respectively [6]. In the long run, shoulder function complications will damage the physical, psychosocial, and social domains of the function of BCS; thus, it is still a matter of concern.

Shoulder function complications of breast cancer are caused primarily by surgery, which causes damage and defect of soft tissues and myofascial restrictions on the upper body. In the effect of scar tissue formation, postsurgical pain, protective posturing, or breast reconstruction, chest wall soft tissue will become tight and short, losing their gliding ability that is relative to shoulder joint [3, 7–9]. Besides, radiotherapy probably causes soft tissue fibrosis and radiation dermatitis, leading to shoulder pain and dysfunction. Other complications such as wound complications, lymphedema, and axillary web syndrome, on the other hand, can also induce shoulder function complications [10–13].

Free range-of-motion upper limb exercise following breast cancer treatment can improve shoulder ROM and decrease chest tightness by moving and stretching the affected arm to restore shoulder joint flexibility and increase scar plasticity [14, 15]. Despite the fact that free range-of-motion upper limb exercise can improve long-term gains in shoulder function without increasing wound complications, there remains underutilization of free range-of-motion upper limb exercise, and quite a few BCS still have a restricted shoulder ROM and report shoulder pain even within 6 months after treatment [16, 17]. Many survivors fear movement after surgery because they fear that the affected arm will be injured. According to the cognitive model of fear of movement injury of Johan Vlaeye, individual fears of injury will lead to fear-avoid behaviors in daily activity [18]. Studies have reported that fear of being injured of the affected arm is one of the common barriers to underutilization of rehabilitation for BCS [16, 17].

Two prospective studies have suggested that mirror therapy (MT) may be a useful tool to improve shoulder function in patients with a restricted shoulder ROM and shoulder pain. MT is a classic rehabilitation therapy based on visual feedback of plane mirrored images. Along the midsagittal plane, placing a mirror between the two limbs, the affected limb is placed behind the mirror, and the unaffected limb faces the mirror. In this way, the reflection of the limb in front of the mirror is always superimposed on the limb hidden behind the mirror so that it could create a visual illusion of the health and intactness of the contralateral limb. When both upper/lower extremities move simultaneously, the individual is required to lean forward slightly to watch and concentrate on the complete reflection of the unaffected limb in the mirror and think the reflection is the move of the affected limb behind the mirror. MT is always a useful rehabilitation technique for patients with nerve injury, such as storks and phantom limb pain [18–21]. In addition, a few studies have reported that MT can improve limb motor function in patients without nerve injury. Altschuler and Hu reported a patient who has a fractured wrist, but no neurovascular impairment can extend her wrist actively after 10 weeks of MT intervention [22]. Rosen and Lundborg showed MT improved a woman’s finger active flexion, whose fingers were bit by a cat. In addition, a patient with rheumatoid arthritis gained good coordination and flexibility of the fingers after 8 weeks of MT intervention [23].

The details of MT intervention on the shoulder of two studies are as follows. Louw et al. asked the patients with shoulder pain and limited ROM to move their shoulder in front of a mirror 10 times for 3 mins; immediate assessment after intervention showed that shoulder flexion ROM has a mean increase of 14.5°, and pain levels averagely decreased to 0.48 [24]. Başkaya et al. conducted a two-arm randomized controlled trial of MT in patients with adhesive capsulitis; immediate measurement after intervention showed that shoulder ROM, VAS score, and the University of California-Los Angeles (UCLA) Shoulder Score of the experimental group improved obviously than that of the control group [25].

The potential mechanism of MT on motor function is using a mirrored image of the intact limb to compensate proprioceptive feedback to a motor command originated by the affected limb; in other words, MT normalizes the efference-afference loop of the sensorimotor brain circuits in a movement process [26, 27], in which, visual information primarily influence the proprioception. Studies in healthy individuals found the position of the hand in front of the mirror can influence the position of the hand behind the mirror. Louw and Başkaya measured the outcomes immediately after the intervention; the results of their studies only showed MT has an influence on shoulder movement. However, whether MT can improve shoulder function in a long-term intervention needs advanced study. Based on current studies, we are interested in the efficacy of MT on shoulder function in BCS. In addition, to our knowledge, no study has applied MT on shoulder function in BCS. What is more, MT has advantages of low cost, practicality, and easy-to-apply; if this trial provides expected results, MT will be expected to promote shoulder rehabilitation of BSC.

## Objectives

The purpose of this prospective, exploratory randomized controlled trial is that if free range-of-motion upper limb exercise based on MT will bring gains on efficacy compared with the effects of free range-of-motion upper limb exercise on shoulder function in patients after surgery of breast cancer. The main purposes of the trial are as follows:

1. Observing the efficacy of free range-of-motion upper limb exercise based on MT on shoulder ROM in BCS
2. Observing the effects of free range-of-motion upper limb exercise based on MT on shoulder function score, shoulder pain, function, and disability of the upper limb in BCS
3. Observing the effects of free range-of-motion upper limb exercise based on MT on fear of movement in BCS.

## Methods

### Study design

**Fig. 1:**
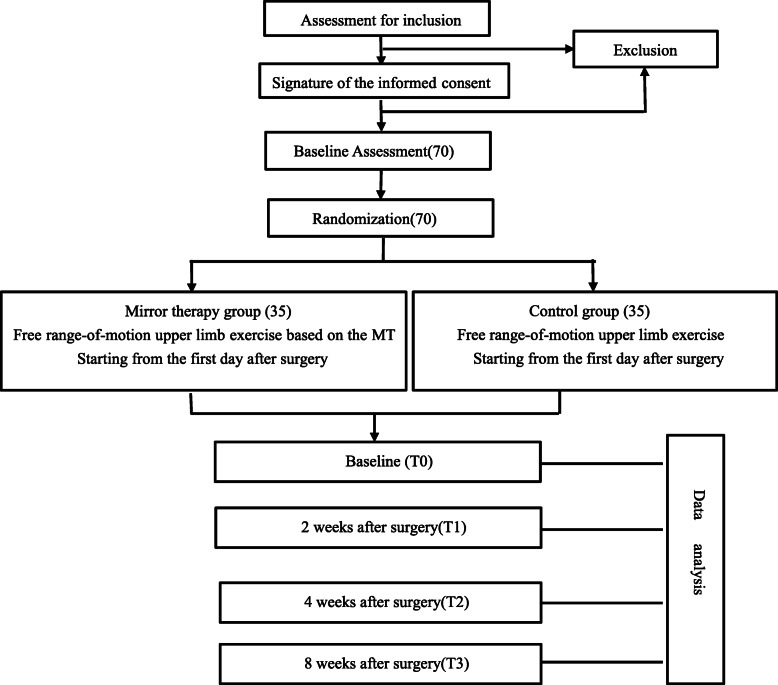
Recruitment and intervention processes flow chart

This is a prospective, single-blind, two-arm randomized controlled trial that will explore the effects of free range-of-motion upper limb exercise based on MT on shoulder function in patients after surgery of breast cancer. An estimated 70 participants from the galactophore department will be randomly allocated to (1) the MT group or (2) the control group. The control group is required to do free range-of-motion upper limb exercise, while the MT group engages in free range-of-motion upper limb exercise based on MT. An intervention team consisting of nurses, rehabilitation therapists, and surgeons will be set up. Outcomes will be measured at baseline and at 2 weeks, 4 weeks, and 8 weeks after the operation. Figure 1 shows the Recommendations for Intervention Trials (SPIRIT) flow diagram for this study.

### Ethical

The ethical approval of this study was approved by the Ethical Committee of Longhua Hospital affiliated to the Shanghai University of Traditional Chinese Medicine on 5 May 2020 (reference number: 2020LCSY016). The research scheme, case report form, and informed consent form were approved by the Ethics Committee. Informed consent will be obtained from all participants before the intervention.

### Study setting

The study will be carried out at the galactophore department in the two hospitals in Shanghai, Longhua Hospital Shanghai University of Traditional Chinese Medicine and Fudan University Zhongshan Hospital. Outcome measurement will be conducted in a separate quiet room in the hospital.

#### Sample size

The primary outcome in this study is shoulder ROM, which is the main effect indicator for sample size estimating in this randomized controlled trial. Referring to Cohen’s effect size value of 1.3799 in the study of Başkaya et al. [25], to achieve 95% power and 0.05 error range, 30 of the total sample size were calculated using the G-power 3.1 software. However, a larger sample size was planned to be used in this trial due to repeated measurement. We used the GLIMMPSE software for repeated measurement calculation with the shoulder flexion range of motion as the main effect indicator that refers to the results of Başkaya et al. [25]. We choose a test power of 0.95 and a type 1 error rate of 0.05, 58 of sample size was calculated, considering 20% dropout rate, the actual total sample size is 70.

### Participants

#### Inclusion criteria

1. Women aged ≥ 18 years.
2. Clinically diagnosed with breast cancer for the first time according to imaging examination and (or) pathological examination.
3. The surgical treatment of breast includes modified radical mastectomy, mastectomy, or conservation surgery; the surgical treatment of the axilla is axillary lymph nodes dissection (ALND) or sentinel lymph node biopsy (SLNB).
4. Having the ability to use social software such as WeChat.
5. The signing of the informed consent form.

#### Exclusion criteria

1. Clinically diagnosed as bilateral breast cancer or cancer metastasis;
2. With shoulder disease, upper limb fracture, neurological deficits, lymphangitis, and any injury of the upper limbs
3. Having suffered from severe cardiovascular disease, cognitive limitations, or mental disease
4. Having visual field defect
5. Receiving immediate reconstruction in surgery

#### Drop out criteria

1. The operation cannot meet the inclusion criteria
2. Withdrawing from trial voluntary
3. Passing away during the trial
4. Losing contacts such as moving residence or changing hospital

### Recruitment

We will recruit eligible participants from 1 July 2020 and complete in June 2021. According to the fixed date of admission of breast cancer patients in two hospitals, we regularly recruit participants at Fudan University Zhongshan Hospital every Monday, and at the other hospital every Thursday. The primary researcher is responsible for the recruitment and can access the medical history of patients after getting permission. Pictures, relevant materials, and informed consent will be used to introduce the program and purpose of the trial, telling patients the advantages and disadvantages of the intervention. We reconfirm and determine the participant of her eligibility according to the inclusion/exclusion criteria. Eligible participants who would have signed the informed consent will finally enter the study.

### Randomization and blinding

Random number sequences are generated in the randomization software by an independent researcher; the 200,000 was used as the seed number to generate 70 random numbers. For avoiding tampering or transillumination, information of trial was written on the two layers of carbonless copy paper, which was placed in sealed, opaque, sequentially numbered envelopes. An independent research assistant will conduct the group allocation. Participants are required to write their name and admission number on the first layer of paper after disclosing the envelopes, random number sequence and group allocation can be seen only on the second layer of paper, and once the two layers of paper are separated, it cannot be restored. After participants sign the informed consent, the rater completes the baseline assessment, then the research assistant who is blinded to the study will perform the group allocation. Eligible participants will be randomly divided into either the MT group or the control group in a ratio of 1:1. After disclosing the envelope, the participant who obtains a paper marked with the number “1” will be allocated to the MT group, while the participant who obtains a paper marked with “2” will be grouped to the control group. Because of the nature of the intervention, rehabilitation therapists and nurses are not blinded, but they are not informed of the trial purpose; the rater, research assistant, and statistician are blinded, and they are also asked not to seek the group allocation and intervention from participants.

### Intervention

Participants are randomly grouped into either the MT group or the control group. Both groups participate in free range-of-motion upper limb exercise from the first day after the operation. Free range-of-motion upper limb exercise is the recommendation of the guidelines and specifications for breast cancer diagnosis and treatment developed by the Chinese Anti-Cancer Association, Committee of Breast Cancer Society (2019 edition) [14]. The control group engages in free range-of-motion upper limb exercise in the usual way, while the MT group receives free range-of-motion upper limb exercise based on MT. The intervention instructions will be provided to participants at the time from the admission to the operation, ensuring they are familiar with their exercise. Both groups are supervised and instructed by nurses and rehabilitation therapists. Upon discharge, each participant will be given an exercise log, which is used to record exercise time and frequency, as well as adverse events. Participants are required to bring the exercise log at every assessment for review. In addition, verbal instruction, a written booklet, and videos designed for the intervention will be provided to the participants for facilitating the intervention at home. What is more, participants in the MT group will be provided with a plane mirror. After discharge, participants are asked to exercise at home and will be supervised by intervention staff every Tuesday or Thursday by WeChat or telephone. For participants with poor adherence, the intervention staff will give them verbal encouragement and motivation and regularly reward participants who adhere to the exercise (e.g., small juicer, vacuum cup, towel, toothpaste). The intervention staff will share health knowledge to both groups biweekly involving diet, self-care, exercise, and sexual life for maintaining the long-term trust of participants in the study. Both groups are asked not to involve in exercises such as arm stretch, yoga, and pilates, but participants who report morning stiffness on their shoulder more than 30 mins are permitted. The elastic bandage is also not allowed in intervention. The intervention in both groups is as follows.

#### Control group

Participants of the control group receive free range-of-motion upper limb exercise from the first day after the operation as shown in Table 1.

**Table 1:** BC Rehabilitation program

**• Preparation phase** 1. If possible, exercise in a quiet room. 2. Do not exercise when you feel dizzy, nauseous, or have lower back pain. 3. If you take blood pressure medication, exercise after 30 minutes. 4. If your shoulder appears morning stiffness more than 30 minutes please perform 5-10 minutes arm stretch training combined with massage before exercise. **• BC Rehabilitation** 1. **1–2 days after surgery** a. Please stand relaxed and hold a bouncy ball in the palm of your hand, put your arm in a position with flexed elbows and slightly extended shoulders, then squeeze the ball as hard as possible for 2-3 seconds, then spread and relax fingers. b. Arms are positioned in the same as above, then rotate the wrist clockwise and anticlockwise. Frequency: 10 repetitions a set, 2 sets a group, 2 groups daily. Efficacy: to promote muscle contraction, blood, and lymph flow, and avoid upper limbs swelling 2. **3-7days after surgery.** a. Please stand relaxed, put your arm by your sides, then touch the ipsilateral and contralateral shoulder alternately by your hand of the affected limb. b. keep the exercise in stage 1. Frequency: 10 repetitions a set, 2 sets a group, 2 groups daily. Efficacy: to recover rotation and adduction. 3. **8-14days after surgery.** a. Please stand, put your arm by your sides, raise the arms forward in a range around 120°, perform backward in a range around 60°. b. Please stand, put your arm in a position with flexed elbows and slightly extended shoulders( neutral ), then perform abduction and adduction of the shoulder(shoulder in adduction). c. Please stand, put your arm by your sides, then perform abduction and adduction of the shoulder in a range around 120° d. ** Turn your shoulders forward and backward e. ** please stand in front of the wall, place your palm of the affected arm on the wall and run up by moving your finger until you feel can’t run up more, maintain for 1mins, then run down. Frequency:10 repeats a set, 3 sets a group, 2 groups daily. Efficacy: to promote shoulder flexion, abduction, and adduction. 4. **2-8weeks after surgery** a. Please stand relaxed, put your arms by your sides, then raise your arms forward and backward to an active range of motion as most tolerated b. Please stand relaxed, place your arms in abduction at shoulder height and flex elbows in 90 degrees( 90° of abducted position), then rotate the forearms up and down. c. Please stand relaxed, put your arm by your sides, then perform abduction and adduction of the shoulders to an active range of motion as most tolerated d. Keep the “d and e” in stage 3. Frequency:10 repeats a set, 3 sets a group, 2 groups daily, 4days per week. Efficacy: to stretch and release the softness in the anterior chest wall to avoid the chest wall tightness. **•Attention!** 1. Please don’t forget the “Preparation phase” before your exercise. 2. It’s better not to arbitrarily change the prearranged sequence of exercise for safety and efficacy. 3. At stages 3 and 4, increase the range of motion as tolerated. 4. A muscular glide in the wrist can be seen when you squeeze the ball. 5. Don't bend the elbow when you perform flexion and abduction of the Shoulder. 6. If the pain after exercise persists, please contact the intervention nurse as soon as possible.	

**The movement will be performed without MT for mirror therapy

#### Mirror group

**Fig. 2:**
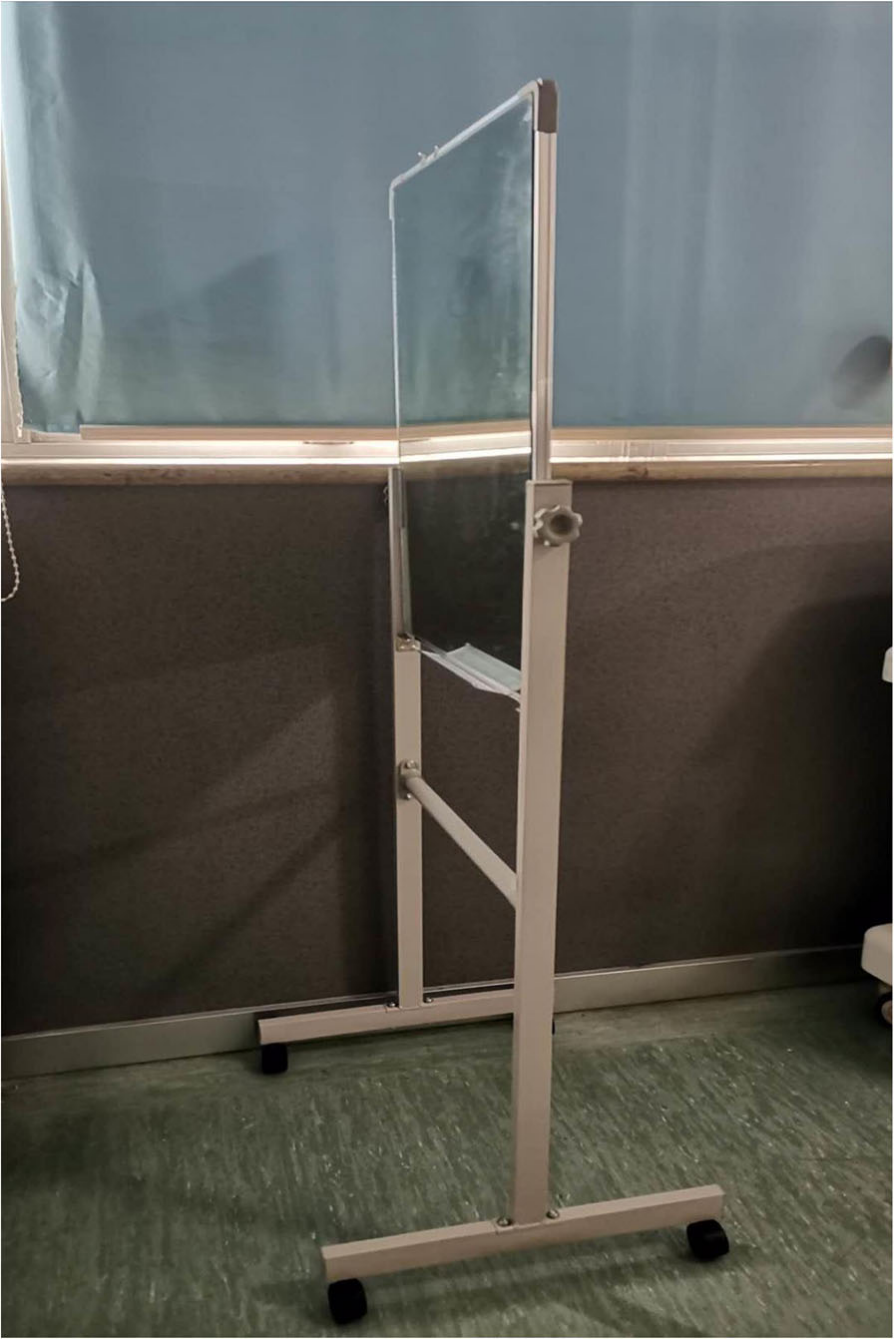
The standing mirror

Participants in the MT group receive free range-of-motion upper limb exercise based on MT from the first day after the operation, the program of free range-of-motion upper limb exercise is the same as the control group. The standing mirror that is designed for this trial consists of a plane mirror, an adjustable bracket, and two wheels; the size of the mirror is 70 cm × 50 cm, and the height of the adjustable bracket is 1.2–2 m (Fig. 2). The MT intervention process is as follows:

1. Preparation: MT will be carried out in a separate, quiet room. Participants are required to remove any accessories (including watches, bracelets, rings, hair bands) on both upper limbs before intervention. Participants choose to sit or stand depending on themselves. Along the midsagittal plane, the standing mirror is placed in front of the participants, the reflective side faces the unaffected limb, and the affected limb is hidden behind the mirror. Participants are asked to lean forward slightly to watch the complete reflection of the unaffected limb in the mirror.
2. Warm-up: Participants will be told to relax and concentrate on the reflection in the mirror and imagine it as the limb on the affected side, and then optionally move the upper limbs for 2~3 min. This process will repeat 3 times.
3. Exercise: The therapist instructs the participants to perform free range-of-motion upper limb exercise as described in Table 1. Participants are asked to watch the reflection in the mirror of their unaffected limb and think of it as their affected upper limb. During the exercise, participants need to concentrate on the reflection in the mirror.

### Potential advert events and exercise adherence

The nurse needs to regularly record the volume of fluid drainage, as well as observe the wound and measure arm circumference. Adverse events include but are not limited to wound drainage volume increases over 50 ml 1 week later after the operation, delayed wound healing, seroma, skin flap necrosis, and persistent shoulder pain. Any adverse event occurring after the entry into this study of participants will be truthfully recorded. The participant who reports adverse events will have the right to withdraw and get free treatment, as well as evaluation. If participants wish to continue the intervention, we still provided exercise to them. Adherence to exercise is deemed to be an attendance of 85% of planned exercise sessions, which is a total of 261 sets. Adherence will be calculated according to the records of the exercise log.

### Outcomes measures

Outcomes will be measured by a trained rater who is blinded to the group allocation at baseline (T0) and at 2 weeks (T1), 4 weeks (T2), and 8 weeks (T3) after the operation. It is necessary to mention that the four time points are not originally registered time points; the reason for the adjustment is as follows. Firstly, the investigation and preliminary experiment were conducted after the originally registered protocol, it was found that the four time points in this protocol are significant points in observing shoulder rehabilitation, compared with originally registered time points, and using these four time points can achieve the aim of measuring shoulder function without increasing workload. Secondly, patients regularly follow up at these time points, which will facilitate the rater of conducting measurement face to face with participants on time. The investigation and preliminary experiment are not within this study, and the change occurred before the study began. Participants’ demographic information and medical history are collected at baseline, and the primary and secondary outcomes are as follows.

#### Primary outcome

To explore the effects of MT on shoulder function, shoulder joint ROM on the affected limb is the primary outcome. An arm goniometer will be used to obtain shoulder ROM measurement including forward flexion, internal rotation, external rotation, and abduction. We follow Struyf and Meeus’ recommendations to conduct the goniometric shoulder joint ROM measurements [28]. Participants sit and put their arms by their sides. Before the assessment, they will be asked to move their shoulders and stretch their arms to relax the muscle, then move the affected arm following the instructions as possible as they can until they feel tired. Active external rotation and internal rotation are measured in 90° of the horizontal abducted position of the shoulder; meanwhile, the rater will press the participants’ acromion of the scapula to avoid a joint compensatory.

#### Secondary outcome

Shoulder function scores will be measured by the Constant-Murley Score(CMS), which is the most used tool with good reliability and validity for shoulder function scores [29]. The instrument evaluates shoulder function scores from four different domains (pain, daily activity, active ROM, and strength), among which, pain and daily activity are self-reported sub-scales, and the other two domains involve objective assessment of shoulder ROM and shoulder abduction strength. The total score of the instrument is 100, a higher score indicates a better shoulder function.

For investigating the fear of movement of subjects with musculoskeletal injury, the Tampa Scale of Kinesiophobia is always considered to be a reliable and valid instrument. There have been studies that used the Tampa Scale of Kinesiophobia on fear of movement in BCS [30–32]. The original Tampa Scale of Kinesiophobia with 17 items was developed in 1991 by Miller, et al. [33], one of its revised versions with 13 items that has comparable reliability and validity to the original version will be used in this study [34]. The 13-item TSK is a self-report questionnaire with scored on a 4-point Likert scale ranging from 1 (strongly disagree) to 4 (strongly agree). The total score of scale ranges between 13 and 52; higher scores represent stronger fear-avoidance beliefs. Hu has verified the validity and reliability of the Chinese version of the TSK with Cronbach α of 0.778 and reliability of 0.860 [35].

The Disabilities of Arm, Shoulder, and Hand Questionnaire (DASH) is a widely used 30-item self-reported questionnaire that will be used to measure the function and symptoms of the affected upper limb in this study. This questionnaire has the level of agreement that is rated on a five-point Likert scale, and higher scores indicate heavier problems in arm functioning or symptoms. The questionnaire assesses the problems in daily functioning from the arm, shoulder, or hand troubles (21 items), abnormal symptoms of the arm (5 items), as well as the effects of arm functional problems on social activities, work, sleep, and psychology (4 items).

Visual analog scale (VAS) is a convenient, simple instrument with a numeric rating scale marked from 0 to 10 that enables patients to mark their pain intensity on the line. Numerical values from 0 to 10 represent the pain intensity from no “pain” to “worst pain” correspondingly. In this trial, we assess the pain of participants both in rest and free range-of-motion upper limb exercise.

Grip strength will be measured with a grip strength meter. Before the test, adjust the unit and years in the display panel. The participant will be asked to sit down in a chair and bend her knee to 90°, then holds the grip force meter and slowly pulls the handle as hard as possible. Participants test twice, selecting the highest value as the final record.

Arm circumference will be assessed by a non-stretch tape at styloid and every 10 cm intervals from the ulnar styloid up to 40 cm distally. The difference of more than 2 cm of arm circumference between the limbs at any measurement point will be considered as lymphedema. Incidence of lymphedema is the proportion of participants who develop lymphedema accounts for total participants.

DASH and TSK are not originally registered outcomes but were identified as observing indicators before the study began. It was found the TSK has already been reported in patients with breast cancer by referring to articles. To improve the study purpose, the TSK was added. DASH can assess the disability and function of the upper limbs. Compared with shoulder ROM, CMS, and grip strength, DASH can observe the effects of the intervention on daily activity ability in BCS; thus, it was also added.

### Data collection and management

The baseline assessment will be complete before the operation. The rater will conduct the second, the third, and the fourth assessment (2 weeks, 4 weeks, and 8 weeks after the operation) at the ward or outpatient where patients will have follow-up visits, facilitating the data collecting. A case report form (CRF) that is identified using participants’ numbers is designed, information of group allocation on it is hidden. The rater is asked to record all information down in CRF truthfully, timely, and accurately. An independent clinical assistant who manages the medical history of participants needs to check the completeness and accuracy of data at each evaluation, to avoid modification, an encrypted phone will be used to take photos of data. We also take effective measures to manage the database. Paper form data will be transcribed in a password-protected computer by two independent data entry personnel using the input review mechanism. The final trial data set will be accessed and analyzed by an independent statistician and primary researcher. The data will be uploaded to the ResMan Raw data sharing platform (IPD sharing platform) of the Chinese Clinical Trial Registry in 6 months upon the completion of the trial. The URL of sharing platform is http://www.medresman.org.cn/login.aspx. The article of this study will be submitted to peer-reviewed journals.

### Trial monitoring and quality control

To assess the fidelity of intervention and improve the quality of the trial, the study will be surveilled and monitored by the Ethical Committee and research center every 4 months; they are independent of the study and have no competing interests. The content surveilled includes the number of eligible participants, data, exercise logs. The primary researcher needs to report the processes and problems of the study and put forward corresponding solutions. An interim analysis is required at the mid-term examination, the primary researcher will submit test summary, CRF, and raw data set to the research center at the final examination.

### Data analysis

Data analysis will be processed by an independent statistician and primary researcher using the SPSS24.0 software. All statistical tests will be double sides; the statistical significance is set at *p* value < .05. Means and standard deviation or median and inter-quartile ranges will be calculated for continuous variables normally distributed and non-normally distributed, respectively. Categorical variables will be described as frequencies (*f*) and percentages (%). Demographics and other baseline values will be analyzed using the independent two-tailed *t*-test, non-parametric Mann-Whitney *U*-test, and chi-square test.

This study will adopt the modified intention-to-treat (mITT) principle. The effects of the intervention on primary and secondary outcomes (excluded arm circumference and incidence of lymphedema) between the groups will be analyzed by generalized estimating equation (GEE) from treatment, time points, and the interaction between group and time. Observed values of those outcomes at four assessments time points will be put into GEE, selecting the auto-correlation matrix and the robust standard error estimation method. Taking outcome index as the response variable, the group, time, and interaction of group and time will be incorporated into the model, in addition, selecting radiotherapy, the dominant hand, and type of surgery as covariates. The model includes the main effect and interaction effect, paired comparison variables will be selected, and the least significant difference (LSD) method will be used to adjust the multiple comparison effect. Arm circumference and incidence of lymphedema will be analyzed at each measurement time point between groups using the independent two-tailed *t*-test and chi-square test. In case of missing data, if missing data is completely random, the sample will not be included in the final data analysis. If missing data is incomplete random missing or non-random missing, multiple interpolations will be used to complete missing values.

## Discussion

This study is a randomized controlled trial, participants in the control group receive free range-of-motion upper limb exercise, and participants in the MT group receive free range-of-motion upper limb exercise based on MT. The trial aims to explore whether MT can bring gains of the efficacy of free range-of-motion upper limb exercise on shoulder function in patients after breast cancer surgery. BCS always complain of shoulder function complications such as a restricted shoulder ROM, shoulder pain, weakness, and stiffness at the shoulder; it adversely affects the physical, psychosocial, and social domains of BCS. Despite breast cancer rehabilitation can prevent shoulder function complications, there seems to be an underutilization of rehabilitation. Recently, a few research have shown that MT has an influence on shoulder movement and pain score in patients with shoulder disease. Up to date, there is only one study that showed MT can relieve the persistent pain in BCS [36], but the effects of MT on shoulder function have not been reported. MT is a bilateral, symmetrical, convenient motor task, free range-of-motion upper limb exercise that involves both upper limbs; given that they have the same performing mode, we will combine MT with free range-of-motion upper limb exercise in this study to observe the effects of the long-term intervention of MT on shoulder function in BCS. If the purpose of this study can be examined, MT may become a potential nursing technique for shoulder function in BCS. We look forward to this randomized controlled trial that can provide a successful combination of nurse and rehabilitation, which will positively impact nurse interdisciplinary research about MT.

## Study status

The recruitment of participants started on 1 July 2020; at the time of the manuscript submission, it is still ongoing. Affected by the epidemic of the new coronavirus, the recruitment is expected to be completed on 31 June 2021, the version number and protocol number: V2.0, 30 April 2021.

## Supplementary Information

**Additional file 1..** Standard Protocol Items: Recommendations for Interventional Trials (SPIRIT) 2013 Checklist [37]: recommended items to address in a clinical trial protocol and related documents.

## Data Availability

Data sharing is not applicable to this article at present. The results of the study will be submitted for publication once the data collection and analysis are completed.

## Abbreviations

BCS: Breast cancer survivors
CMS: Constant-Murley Score
CRF: Case report form
DASH: Disability of the Arm, Shoulder, and Hand
MT: Mirror therapy
ROM: Range of motion
TSK: Tampa Scale of Kinesiophobia
VAS: Visual analog scale
