# Supplementary material for: Effects of free range-of-motion upper limb exercise based on mirror therapy on shoulder function in patients after breast cancer surgery: study protocol for a randomized controlled trial
Source: Trials. 2021 Nov 17;22:815. doi: 10.1186/s13063-021-05789-2 (PMC8596341; doi:10.1186/s13063-021-05789-2)
Supplement: Supplementary file 1 — Additional file 1.. Standard Protocol Items: Recommendations for Interventional Trials (SPIRIT) 2013 Checklist [37]: recommended items to address in a clinical trial protocol and related documents. [file 13063_2021_5789_MOESM1_ESM.doc]

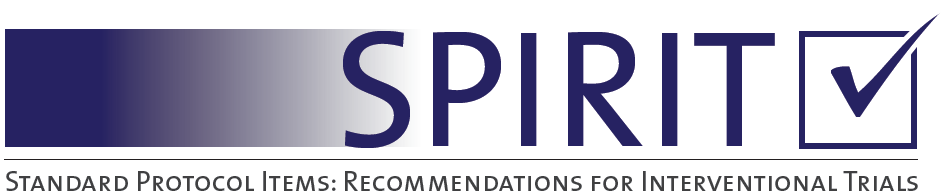


SPIRIT 2013 Checklist: Recommended items to address in a clinical trial protocol and related documents*

| Section/item | ItemNo | Description Page/paragraph |
| --- | --- | --- |
| **Administrative information** | | |
| Title | 1 | Descriptive title identifying the study design, population, interventions, and, 1/3  if applicable, trial acronym |
| Trial registration | 2a | Trial identifier and registry name. If not yet registered, name of intended registry 3/55 |
|  | 2b | All items from the World Health Organization Trial Registration Data Set N/A |
| Protocol version | 3 | Date and version identifier 19/394 |
| Funding | 4 | Sources and types of financial, material, and other support 20/403 |
| Roles and responsibilities | 5a | Names, affiliations, and roles of protocol contributors 20/407 |
|  | 5b | Name and contact information for the trial sponsor 20/417 |
|  | 5c | Role of study sponsor and funders, if any, in study design; collection, management, 20/403  analysis, and interpretation of data; writing of the report; and the decision to submit  the report for publication, including whether they will have ultimate authority over any  of these activities |
|  | 5d | Composition, roles, and responsibilities of the coordinating centre, steering committee, 17/345  endpoint adjudication committee, data management team, and other individuals or groups  overseeing the trial, if applicable (see Item 21a for data monitoring committee) |
| Introduction |  |  |
| Background and rationale | 6a | Description of research question and justification for undertaking the trial, including 2-7  summary of relevant studies (published and unpublished) examining benefits and  harms for each intervention |
|  | 6b | Explanation for choice of comparators 4/83 |
| Objectives | 7 | Specific objectives or hypotheses 6/128 |
| Trial design | 8 | Description of trial design including type of trial 7/139  (eg, parallel group, crossover, factorial, single group),  allocation ratio, and framework  (eg, superiority, equivalence, noninferiority, exploratory) |
| Methods: Participants, interventions, and outcomes | | |
| Study setting | 9 | Description of study settings (eg, community clinic, academic hospital) and list of 7/153  countries where data will be collected. Reference to where list of study  sites can be obtained |
| Eligibility criteria | 10 | Inclusion and exclusion criteria for participants. If applicable, eligibility criteria 8/166  for study centres and individuals who will perform the interventions  (eg, surgeons, psychotherapists) |
| Interventions | 11a | Interventions for each group with sufficient detail to allow replication, 10/213  including how and when they will be administered |
|  | 11b | Criteria for discontinuing or modifying allocated interventions for N/A  a given trial participant (eg, drug dose change in response to harms,  participant request, or improving/worsening disease) |
|  | 11c | Strategies to improve adherence to intervention protocols, and any 11/221-231  procedures for monitoring adherence (eg, drug tablet return, laboratory tests) |
|  | 11d | Relevant concomitant care and interventions that are permitted 11/232  or prohibited during the trial |
| Outcomes | 12 | Primary, secondary, and other outcomes, including the specific measurement 14/269  variable (eg, systolic blood pressure), analysis metric (eg, change from baseline,  final value, time to event), method of aggregation (eg, median, proportion),  and time point for each outcome. Explanation of the clinical relevance  of chosen efficacy and harm outcomes is strongly recommended |
| Participant timeline | 13 | Time schedule of enrolment, interventions (including any run-ins and washouts), Fig.1  assessments, and visits for participants. A schematic diagram is highly  recommended (see Figure) |
| Sample size | 14 | Estimated number of participants needed to achieve study objectives and how 8/157  it was determined, including clinical and statistical assumptions supporting  any sample size calculations |
| Recruitment | 15 | Strategies for achieving adequate participant enrolment to reach target sample size 9/188 |
| **Methods: Assignment of interventions (for controlled trials)** | | |
| Allocation: |  |  |
| Sequence generation | 16a | Method of generating the allocation sequence (eg, computer-generated 9/198  random numbers), and list of any factors for stratification. To reduce  predictability of a random sequence, details of any planned restriction  (eg, blocking) should be provided in a separate document that is unavailable  to those who enrol participants or assign interventions |
| Allocation concealment mechanism | 16b | Mechanism of implementing the allocation sequence (eg, central telephone; 10/200  sequentially numbered, opaque, sealed envelopes), describing any steps to  conceal the sequence until interventions are assigned |
| Implementation | 16c | Who will generate the allocation sequence, who will enrol participants, and 9/198  who will assign participants to interventions |
| Blinding (masking) | 17a | Who will be blinded after assignment to interventions (eg, trial participants, 10/209  care providers, outcome assessors, data analysts), and how |
|  | 17b | If blinded, circumstances under which unblinding is permissible, and N/A  procedure for revealing a participant’s allocated intervention during the trial |
| **Methods: Data collection, management, and analysis** | | |
| Data collection methods | 18a | Plans for assessment and collection of outcome, baseline, and other trial data, 17/330  including any related processes to promote data quality (eg, duplicate  measurements, training of assessors) and a description of study instruments  (eg, questionnaires, laboratory tests) along with their reliability and validity,  if known. Reference to where data collection forms can be found, if not in the protocol |
|  | 18b | Plans to promote participant retention and complete follow-up, including list 11/221-230  of any outcome data to be collected for participants who discontinue or  deviate from intervention protocols |
| Data management | 19 | Plans for data entry, coding, security, and storage, including any related 17/333-340  processes to promote data quality (eg, double data entry; range checks  for data values). Reference to where details of data management  procedures can be found, if not in the protocol |
| Statistical methods | 20a | Statistical methods for analysing primary and secondary outcomes. 18/352  Reference to where other details of the statistical analysis plan can  be found, if not in the protocol |
|  | 20b | Methods for any additional analyses (eg, subgroup and adjusted analyses) N/A |
|  | 20c | Definition of analysis population relating to protocol non-adherence 18/352  (eg, as randomised analysis), and any statistical methods to  handle missing data (eg, multiple imputation) |
| **Methods: Monitoring** | | |
| Data monitoring | 21a | Composition of data monitoring committee (DMC); summary of its role 17/330  and reporting structure; statement of whether it is independent from the  sponsor and competing interests; and reference to where further details  about its charter can be found, if not in the protocol. Alternatively,  an explanation of why a DMC is not needed |
|  | 21b | Description of any interim analyses and stopping guidelines, 18/349  including who will have access to these interim results and  make the final decision to terminate the trial |
| Harms | 22 | Plans for collecting, assessing, reporting, and managing solicited 14/260  and spontaneously reported adverse events and other unintended  effects of trial interventions or trial conduct |
| Auditing | 23 | Frequency and procedures for auditing trial conduct, if any, and whether 18/345  the process will be independent from investigators and the sponsor |
| Ethics and dissemination | | |
| Research ethics approval | 24 | Plans for seeking research ethics committee/institutional review board 7/148  (REC/IRB) approval |
| Protocol amendments | 25 | Plans for communicating important protocol modifications (eg, changes to N/A  eligibility criteria, outcomes, analyses) to relevant parties (eg, investigators,  REC/IRBs, trial participants, trial registries, journals, regulators) |
| Consent or assent | 26a | Who will obtain informed consent or assent from potential trial participants 7/152+9/192  or authorised surrogates, and how (see Item 32) |
|  | 26b | Additional consent provisions for collection and use of participant data and N/A  biological specimens in ancillary studies, if applicable |
| Confidentiality | 27 | How personal information about potential and enrolled participants will be 9/192+17/333  collected, shared, and maintained in order to protect confidentiality before,  during, and after the trial |
| Declaration of interests | 28 | Financial and other competing interests for principal investigators for the 20/402  overall trial and each study site |
| Access to data | 29 | Statement of who will have access to the final trial dataset, and disclosure 17/340  of contractual agreements that limit such access for investigators |
| Ancillary and post-trial care | 30 | Provisions, if any, for ancillary and post-trial care, and for compensation to 14/265  those who suffer harm from trial participation |
| Dissemination policy | 31a | Plans for investigators and sponsor to communicate trial results to participants, 17/341  healthcare professionals, the public, and other relevant groups (eg, via publication,  reporting in results databases, or other data sharing arrangements), including  any publication restrictions |
|  | 31b | Authorship eligibility guidelines and any intended use of professional writers N/A |
|  | 31c | Plans, if any, for granting public access to the full protocol, participant-level 17/343  dataset, and statistical code |
| Appendices |  |  |
| Informed consent materials | 32 | Model consent form and other related documentation given to participants 21/416  and authorised surrogates |
| Biological specimens | 33 | Plans for collection, laboratory evaluation, and storage of biological specimens N/A  for genetic or molecular analysis in the current trial and for future use in ancillary  studies, if applicable |

*It is strongly recommended that this checklist be read in conjunction with the SPIRIT 2013 Explanation & Elaboration for important clarification on the items. Amendments to the protocol should be tracked and dated. The SPIRIT checklist is copyrighted by the SPIRIT Group under the Creative Commons “[Attribution-NonCommercial-NoDerivs 3.0 Unported](http://www.creativecommons.org/licenses/by-nc-nd/3.0/)” license.
